# Chemical profile and antioxidant capacity verification of Psidium guajava (Myrtaceae) fruits at different stages of maturation

**DOI:** 10.17179/excli2015-522

**Published:** 2015-09-07

**Authors:** Heverton M. Araújo, Fabíola F. G. Rodrigues, Wégila D. Costa, Carla de F. A. Nonato, Fábio F. G. Rodrigues, Aline A. Boligon, Margareth L. Athayde, José G. M. Costa

**Affiliations:** 1 Department of Biological Chemistry, Laboratory of Research in Natural Products, Program of Post-Graduation in Molecular Bioprospection, Regional University of Cariri, 63105-000 Crato, CE, Brazil; 2 Program of Post-Graduation in Biological Sciences-Biochemical Toxicology, Federal University of Santa Maria, Campus Camobi, Santa Maria, RS, 97105-900, Brazil

**Keywords:** Psidium guajava, flavonoids, phenolic compounds, antioxidant activity, HPLC

## Abstract

Psidium guajava (Myrtaceae), a common plant in Cariri region, Ceara, Brazil, as well as in various parts of the world, contains high concentrations of bioactive compounds and in many communities its parts are used for therapeutic purposes. Studies describe antioxidant, antimicrobial and anti-diarrheal actions from extracts obtained from leaves, but information about the activities of the fruits and comparison of these at different maturity stages (immature, partially mature and mature) are scarce. This study aims to evaluate the antioxidant properties by quantifying the levels of phenolic and flavonoid compounds, carotenoids and vitamin C of P. guajava fruits at different stages of maturation. The content of phenolic compounds for the immature fruit, partially mature and mature were: 22.41; 34.61 and 32.92 mg of AG/g fraction. The flavonoid content for immature fruits, intermediate and mature were: 2.83; 5.10 and 5.65 mg RUT/g fraction, respectively. Following the same standards of maturation stages, the ascorbic acid content was determined with values of 0.48; 0.38 and 0.21 mg AA/g fraction, respectively. HPLC analysis identified and quantified the presence of gallic acid, catechin, chlorogenic acid, caffeic acid, epicatechin, rutin, quercitrin, isoquercitrin, quercetin, kaempferol, glycosylated campeferol, tocopherol, β-carotene and lycopene. The antioxidant activity carried out by DPPH method showed the mature fruits bearing the best results, whereas chelation of Fe2+ ions showed higher percentage for the immature fruit. The results obtained by lipidic peroxidation were not satisfactory.

## Introduction

Neurodegenerative diseases exemplified by: Parkinson's, Multiple Sclerosis and Alzheimer's are highlighted by the fact that their etiologies relate to an abnormal production of highly reactive compounds, the free radicals (Bianchi and Antunes, 1999[4]; Ferreira and Matsubara, 1997[13]).

In aerobic organisms, oxidation reactions involved in vital processes such as obtaining energy, phagocytosis, regulation of cellular growth and signaling and synthesis of important substances naturally produce free radicals. The free radical concept is based on electron distribution, being understood as an atom or molecule that has unpaired electrons. In order to highlight the damaging free radicals and add the chemical species that do not have unpaired electrons, the term "reactive species" has been introduced, and it has since been more used than the expression "free radicals" (Halliwell and Guttenridge, 1984[18]; Ribeiro et al., 2005[32]).

Biologically, the reactive oxygen species (ROS) are the most important. At low concentrations they are involved in cell proliferation, biosynthesis, chemotaxis, cell respiration, apoptosis and defense against invading microorganisms. All these events require energy to happen and it is precisely in achieving this that the ROS is formed in the mitochondria (Fridovich, 1999[15]; Oktyabrsky and Smirnova, 2007[27]).

To maintain homeostasis, humans have a defense system against free radicals formed mainly by enzymes and endogenous substances, able to neutralize them preventing the development of its damaging effects. Although effective, this system is not complete and needs supplementation of exogenous antioxidants derived from alimentation, especially products derived from plants. (Fanhani and Ferreira, 2006[12]; Ferreira and Matsubara, 1997[13]; Pereira et al., 2009[28]).

The Myrtaceae family stands out among all botanical families for its scope, possessing more than three thousand species grouped into about 140 genera widespread throughout the world, covering mainly areas of tropical, subtropical and temperate climate (Wilson et al., 2001[39]).

The species of greatest importance in the Myrtaceae family is the *Psidium guajava*, popularly known as guava tree (Franzon et al., 2009[14]). Great economic importance is attributed to this plant in many countries. Its fruits are eaten fresh or in the form of sweets, juices, ice cream, etc. In folk medicine, its leaves are used to treat dysentery and as a cicatrizant. Studies with extracts of the leaves have proven antioxidant, antimicrobial and antidiarrheal activities as well as studies with fruit extracts confirmed hypoallergenic activity (Campos, 2010[8]).

This study aims to evaluate the antioxidant properties by quantifying the levels of phenolic compounds, flavonoids, carotenoids and vitamin C of *P*. *guajava* fruits at different stages of maturation.

## Materials and Methods

### Plant material

*Psidium guajava* fruits at three maturity stages (immature, partially immature and mature) were collected in October 2012 at the Federal Institute of Education, Science and Technology of Ceara, Km 15, city of Crato, CE-Brazil, under the coordinates 7°20'52.28"S, 39°44'79.51"W. A voucher specimen was prepared and deposited under the registration number 9482 in the Dárdano de Andrade Lima Herbarium of the Regional University of Cariri-URCA.

### Preparation of extracts

The fruits were peeled, pulps removed (392.54 g of immature fruits; 603.28 g of partially mature fruits and 596.86 g of mature fruits), and milled with distilled water to prepare the aqueous extract. The yields after lyophilization were: immature pulp (55.97 g; 14.26 %), partially mature pulp (61.05 g; 10.12 %) and mature pulp (59.56 g; 9.98 %).

To obtain the methanolic fractions the lyophilized aqueous extracts (5 g of each maturity stage) were fractionated with methanol. The ethanol was removed using a rotary vacuum evaporator (Model Q-344B, Quimis, Brazil) and an ultra-thermal bath (Model Q-214M2, Quimis) under room temperature and reduced pressure. The yields to the lyophilized extracts were: immature (1.45 g; 29.1 %), partially mature (1:34 g; 26.73 %) and mature (1.24 g; 24.84 %).

### Chemical analysis

#### Chemicals

The chemicals used were of analytical grade. Tris-HCl, 1-1-diphenyl-2-pycrylhydrazyl (DPPH), thiobarbituric acid (TBA), malonaldehyde bis-dimethyl acetal (MDA), trichloroacetic acid (TCA), quercetin, rutin and phenanthroline were obtained from Sigma (St. Louis, MO, USA). Methanol (HPLC grade), ethanol, iron(II)sulphate, gallic, caffeic and chlorogenic acids were purchased from Merck (Rio de Janeiro, Brazil). The water used was purified in Milli-Q plus system from Millipore (Milford, MA, USA). The membranefilter (PRFE 0:45 mm) was obtained from Waters Co. (Milford, MA, USA).

#### pH and acidity

The determination of pH was performed using the potentiometric method under the rules of the Instituto Adolfo Lutz (1985[20]) and the total acidity was carried out according to titrimetric analysis using NaOH solution 0.1 M (Helrich, 1990[19]).

#### Total phenols

The concentration of total phenols was determined by the spectrophotometric method based on procedures described by Singleton et al. (1999[35]). Varying concentrations of methanolic fractions were mixed with 200 of Folin-Ciocalteu solution 10 % and 400 uL of 7.5 % sodium carbonate solution. The mixture was incubated at 45 °C and reacted protected from light for 15 minutes. The blank test was simultaneously prepared using distilled water instead of the samples. The absorbance was determined in spectrophotometer with wavelength adjusted to 765 nm. The same procedure was repeated for the gallic acid used as standard for comparison of phenolic compounds.

#### Total flavonoids 

The total concentration of flavonoids was determined by the spectrophotometric method of aluminum chloride based on procedures described by Venkatachalam et al. (2012[37]). Concentration of the methanolic fractions were prepared in distilled water and mixed with 40 uL of sodium acetate, 0.1 M solution and 40 uL of a 10 % solution of aluminum chloride. The mixture was incubated at room temperature for 45 minutes and reacted protected from light. The blank test was prepared concurrently by using distilled water instead of the samples. The absorbance was measured at wavelength of 415 nm. The same procedure was repeated to obtain the standard curve with rutin.

#### Ascorbic acid 

The concentration of ascorbic acid was determined by the spectrophotometric method described in Leme Junior and Malavolta (1950[24]). 10 grams of lyophilized pulp in the three stages of maturation were immersed in the oxalic acid solution at 2 %. After 24 hours of extraction, the samples were centrifuged and the supernatant was collected for test continuity. Increasing amounts of the supernatant were added in the test tube (200 μL, 400 μL, 600 μL, 800 μL, and 1000 μL) and supplemented with oxalic acid solution to form a final volume of 1000 μL. The absorbance was measured at wavelength of 418 nm 20 seconds after adding 2000 μL dye solution of 2,6-diclorobenzenoindofenol 0.25 mg/mL. The blank test was simultaneously prepared using oxalic acid and the dye solution. The entire test was conducted at room temperature and protected from light. The same procedure was repeated to obtain the standard curve with ascorbic acid.

#### Quantification of phenolics and flavonoids compounds by HPLC-DAD

Reverse phase chromatographic analyses were carried out under gradient conditions using C_18 _column (4.6 mm x 250 mm) packed with 5 μm diameter particles; the mobile phase was water containing 2 % acetic acid (A) and methanol (B), and the composition gradient was: 5 % (B) for 2 min; 25 % (B) until 10 min; 40, 50, 60, 70 and 80 % (B) every 10 min; following the method described by Sabir et al*.* (2012[33]) with slight modifications. The extract of *Psidum guajava *(HM, HDV and HV) and mobile phase were filtered through 0.45 μm membrane filter (Millipore) and then degassed by ultrasonic bath prior to use, the extracts of *Psidum guajava *were analyzed at a concentration of 20 mg/mL. The flow rate was 0.8 mL/min and the injection volume was 50 μL. The sample and mobile phase were filtered through 0.45 μm membrane filter (Millipore) and then degassed by ultrasonic bath prior to use. Stock solutions of standard references were prepared in the HPLC mobile phase at a concentration range of 0.030 - 0.250 mg/mL catechin, epicatechin, quercetin, quercitrin, isoquercitrin, kaempferol and rutin, and 0.045 - 0.500 mg/mL for gallic, caffeic and chlorogenic acids. Quantification was carried out by integration of the peaks using the external standard method, at 257 nm for gallic acid, 280 nm for catechin and epicatechin, 325 nm for chlorogenic and caffeic acids, and 365 for quercetin, quercitrin, isoquercitrin, kaempferol and rutin. The chromatography peaks were confirmed by comparing its retention time with those of reference standards and by DAD spectra (200 to 600 nm). Calibration curve for gallic acid: Y = 13591x + 1187.5 (r = 0.9996); caffeic acid: Y = 11748x + 1340.3 (r = 0.9997); chlorogenic acid: Y = 12685x + 1319.4 (r = 0.9998); catechin: Y = 12547x + 1337.4 (r = 0.9993); epicatechin: Y = 11958x + 1329.7 (r = 0.9990); rutin: Y = 12756x + 1163.8 (r = 0.9991); quercetin: Y = 11891x + 1241.6 (r = 0.9985); quercitrin: Y = 12694x + 1351.0 (r = 0.9989), isoquercitrin: Y = 13079x + 1451.6 (r = 0.9991) and kaempferol: Y = 13657x + 1341.8 (r = 0.9999). All chromatography operations were carried out at room temperature and in triplicate.

#### Quantification of carotenoids by HPLC-DAD 

Carotenoid analysis was performed at reverse phase chromatographic analysis carried out under gradient conditions using C_18 _column (4.6 mm x 150 mm) packed with 5 μm diameter particles. The mobile phase consisted of mixtures of ACN: H_2_O (9:1, v/v) with 0.25 % triethylamine (A) and EtAc with 0.25 % triethylamine (B). The gradient started with 90 % A at 0 min to 50 % A at 10 min. The percentage of A decreased from 50 % at 10 min to 10 % A at 20 min. The flow-rate was 0.8 mL/min and the injection volume was 40 μL. Signals were detected at 450 nm, following the method described by Janovik et al. (2012[22]) with slight modifications. Solutions of standards references (tocopherol, licopene and β-carotene) were prepared in HPLC mobile phase at a concentration range of 0.035 - 0.350 mg/mL. *P. guajava *extracts (HM, HDV and HV) were analyzed at a concentration of 10 mg/mL, carotenoids were identified and quantified in the extracts of *P. guajava* by comparison of retention times and UV spectra with the standard solution. All chromatography operations were carried out at room temperature and in triplicate.

#### Limit of detection (LOD) and limit of quantification (LOQ)

LOD and LOQ were calculated based on the standard deviation of the responses and the slope using three independent analytical curves, as defined by Boligon et al. (2012[5]). LOD and LOQ were calculated as 3.3 and 10 σ/S, respectively, where σ is the standard deviation of the response and S is the slope of the calibration curve.

### Antioxidant activity

#### DPPH radical-scavenging activity

The antioxidant capacity of the methanolic fractions was determined by spectrophotometric method of DPPH radical kidnapping according to Mensor et al. (2001[25]). Fractions of methanolic solutions were prepared in concentrations (µg/mL) of 250, 100, 50, 25, 10, 5 and 2.5 using methanol as solvent. The solutions were mixed with 1500 µL of 0.3 mM DPPH solution and incubated at room temperature for 30 minutes and reacted protected from light. The blank test was simultaneously prepared using water and methanol P.A instead of samples. The absorbance was measured at a wavelength of 517 nm. The same procedure was repeated to obtain the antioxidant capacity of ascorbic acid which was used as positive control.

#### Iron chelation assay

The ability of the methanolic fractions extracted from *P. guajava *fruits to chelate Fe^2+^ ions was determined using the method described by Puntel et al. (2005[30]) with some modifications made. The test was performed with the addition of 150 µL of ferrous sulfate (FeSO_4_) 2 mM in a reaction mixture containing 168 µL of 0.1 M Tris-HCl (pH 7.4), 218 µL saline solutions and the samples with concentrations (µg/mL) of 250, 100, 50, 25, 10, 5 and using distilled water as the solvent. The reaction mixture was incubated, protected from light, for 5 minutes before the addition of 13 µL of 1,1-O-phenanthroline 0.25 %.

Two blank tests were performed in which the first contained the samples solutions in the same concentrations used in the assay and the solution of 1,1-O-phenanthroline 0.25 %. Second, contained the samples solutions in the same concentrations of the samples used in the test and the FeSO_4_ solution also used in the test. The absorbance was subsequently measured at 510 nm on a spectrophotometer and the chelating ability of the samples was expressed as percentage (%) (Batool et al., 2010[3]).

#### Determination of the inhibition of lipid peroxidation

The lipid peroxidation was determined by the measurement of products from the oxidation of lipids that react with thiobarbituric acid (TBARS) between them, the malondialdehyde (MDA). The methodology used in this research uses as a parameter the reading of the solutions absorbance containing the extracts, phospholipids and thiobarbituric acid.

The phospholipids were obtained from egg yolk. A egg yolk solution was prepared (1g: 10 mL) of a solvent mixture containing hexane and isopropyl alcohol at a ratio of 3:2, respectively (Puntel et al., 2007[31]). TBARS production was determined using a modified method of Ohkawa et al., (1979[26]), The methanolic solutions of the fractions of *P. guajava* pulps at concentrations (mg/ mL) of 250, 100, 50, 25, 10 were mixed together with a solution of egg phospholipids and deionized water (total volume of 500 µL) and pre-incubated for 1 hour in a temperature of 37 °C. After this period, 500 µL of acetic acid buffer and 500 µL of 0.6 % TBA were added to the reaction mixture and taken for further incubation at 100 °C for 1 hour. After completion of the incubation, 1.5 mL of butanol was added and the mixture was subjected to centrifugation at 2000 rpm for 2 minutes. The standard curve followed the same procedure as the test being withdrawn the phospholipids solution and the solutions of the methanolic fractions, those replaced by a MDA solution 320 µL. The absorbance was measured at a wavelength of 532 nm.

## Results and Discussion

### Chemical profile

Immature fruits showed dark green peel, rigid consistency and pulp predominantly white. For intermediate fruits, skin color had lighter shades of green and decreased stiffness in relation to the immature fruit and pink pulp. In the mature stage, the peel showed yellow color and soft texture to the touch, with soft consistency and fragile appearance, the pulp showed red color and a pleasant odor that differs from the more immature stages that do not have such a feature.

The observed changes in the three stages of maturation derive from metabolic processes that involve the maturation of the fruits, this is characterized by the autocatalytic production of ethylene, which acts as the signaling molecule inducing the increased synthesis of enzymes. The change in color of the peel is determined by the concentration of chlorophyll. Immature fruits have a higher amount of chlorophyll which decreases with maturity as well as the staining intensity, changing to yellow in the mature fruit. The color change of the pulps is associated with enzyme activity with lycopene production which increases during ripening. The rigidity of the fruits is attributable to the presence of pectins present in the cell wall. During ripening there is an increase in the synthesis of polygalacturonase and pectin methylesterase, enzymes that act solubilizing pectins thus determining the decrease in stiffness (Azzaroli et al., 2004[1]; Brady, 1987[6]; Pereira et al., 2006[29]).

An increase in pH and in titrable acidity was observed during ripening (Table 1(Tab. 1)). These results are similar to those found by Yusof et al. (1988[40]) and Bashir et al. (2003[2]) in their studies. Lee et al. (2010[23]) lists the malic and citric acids, quite influential present in the acidity of the fruit. Citric acid is the major component of all organic acids and the reduction of their concentration during ripening, associated with increased concentration of sugars (galactose and fructose), is related to the increased acidity presented by fruits.

Comparing the results of the levels of phenolic compounds it can be seen that the partially mature and mature stages were not significantly different (Table 1(Tab. 1)). The same observation is made when comparing the same stages in the flavonoid content. Bashir et al. (2003[2]) disagrees with the values and show a decrease in phenolic content during fruit ripening. Also according to Bashir et al. (2003[2]), the amounts of phenolic compounds are higher in the peel if compared to the pulps alone and the variation of concentration of the pink colored pulp was small and in the same range of values obtained in this work.

The immature stage had the lowest levels for phenols and flavonoids (Table 1(Tab. 1)), the result is similar to that found in the hydroalcoholic extract by Vieira et al. (2011[38]). Among the tests, the concentration of the phenols is well above the flavonoids. The literature places the class of flavonoids as a participant of the diverse group of phenolic compounds which also comprises simple phenols, phenolic acids, coumarins, stilbenes, tannins, lignans, lignins and others (Bravo, 1998[7], Croft 1998[10]), therefore, the comparison of the obtained concentrations suggests that there is presence of other phenolic compounds aside from flavonoids, and could not be identified in the tests.

Contrary to what was observed with the content of phenols and flavonoids, the concentrartion of ascorbic acid reaches higher values in unripe fruits decreasing as the fruit ripens (Table 1(Tab. 1)). Bashir et al. (2003[2]) shows values similar to ascorbic acid content during maturation as well as higher values found in the peel regarding the pulp of the fruit.

The molecular content the secondary metabolites does vary and is directly influenced by maturation stage, cultivation, geographical origin, harvest conditions, methodology and solvents used in the extraction (Vieira et al., 2011[38]). Fresh fruit samples have a higher amount of phenolic compounds after extraction than fruits frozen for later lyophilization. During freezing, the cells of the fruit can undergo a joining process of the compartment by breakage of the internal structure culminating in the approach of enzymes and activators of phenolic compounds causing its tdegradation (Shofian et al., 2011[34]).

**Figure 1:**
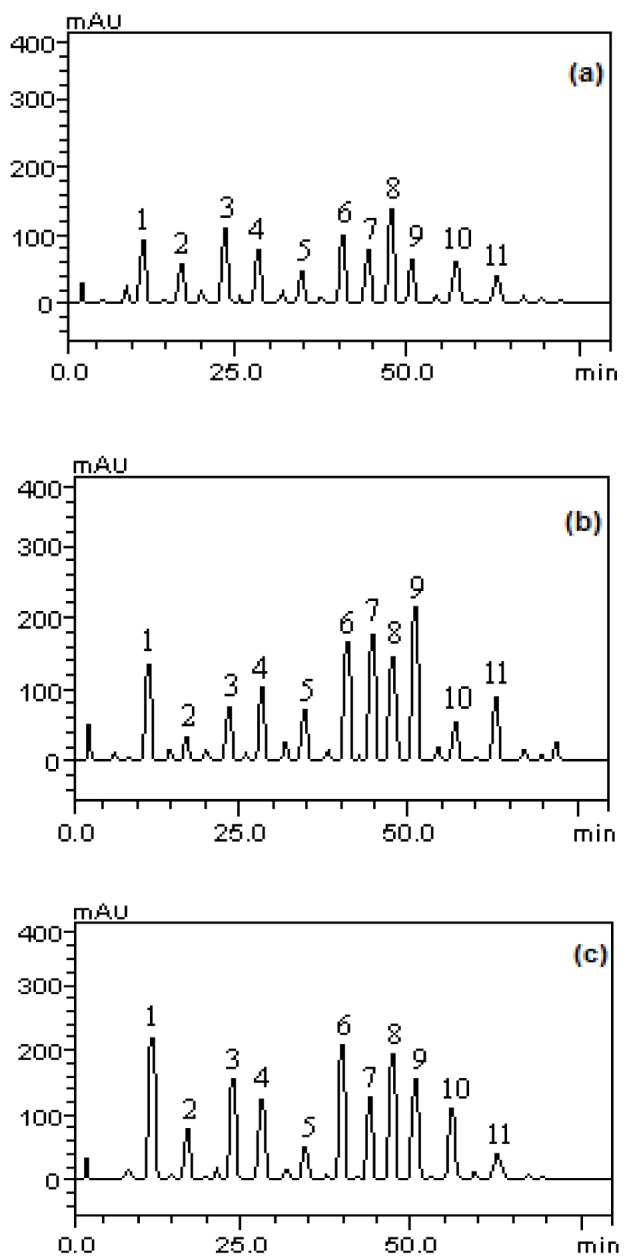
Analysis profile of methanolic fractions of *P. guajava* fruits in immature (a), partially immature (b) and mature stages (c). Gallic acid (peak 1), catechin (peak 2), chlorogenic acid (peak 3), caffeic acid (peak 4), epicatechin (peak 5), rutin (peak 6), quercitrin (peak 7), isoquercitrin (peak 8) , quercetin (peak 9), kaempferol (peak 10) and glycosylated campeferol (peak 11).

The analytical profile of the methanolic fractions of *P. guajava* fruits in immature, partially immature and mature stages to phenols and flavonoids showed the presence of gallic acid (tR = 12.65 min, peak 1), catechin (tR = 16.79 min, peak 2), chlorogenic acid (tR = 24.83 min; peak 3), caffeic acid (tR = 28.05 min; peak 4), epicatechin (tR = 34.96 min; peak 5), rutin (tR = 40.01 min, peak 6), quercitrin (tR = 44.27 min, peak 7), isoquercitrin (tR = 46.85 min, peak 8), quercetin (tR = 50.13 min; peak 9), kaempferol (tR = 55.23 min, peak 10) and glycosylated kaempferol (tR = 63.47 min; peak 11), as well as other components in low concentration (Figure 1(Fig. 1)).

**Figure 2:**
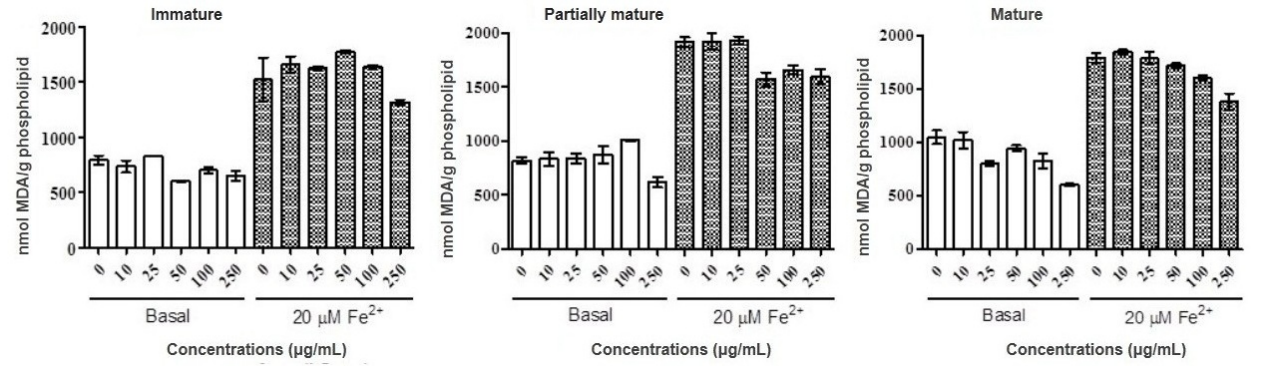
Antioxidant property of methanolic fractions of green maturity, intermediate and mature stages of *Psidium guajava* fruits. Lipid peroxidation using egg phospholipid was determined in the presence and absence of Fe^2+^ 20μM. Results are expressed as mean ± SD of three determinations.

**Figure 3:**
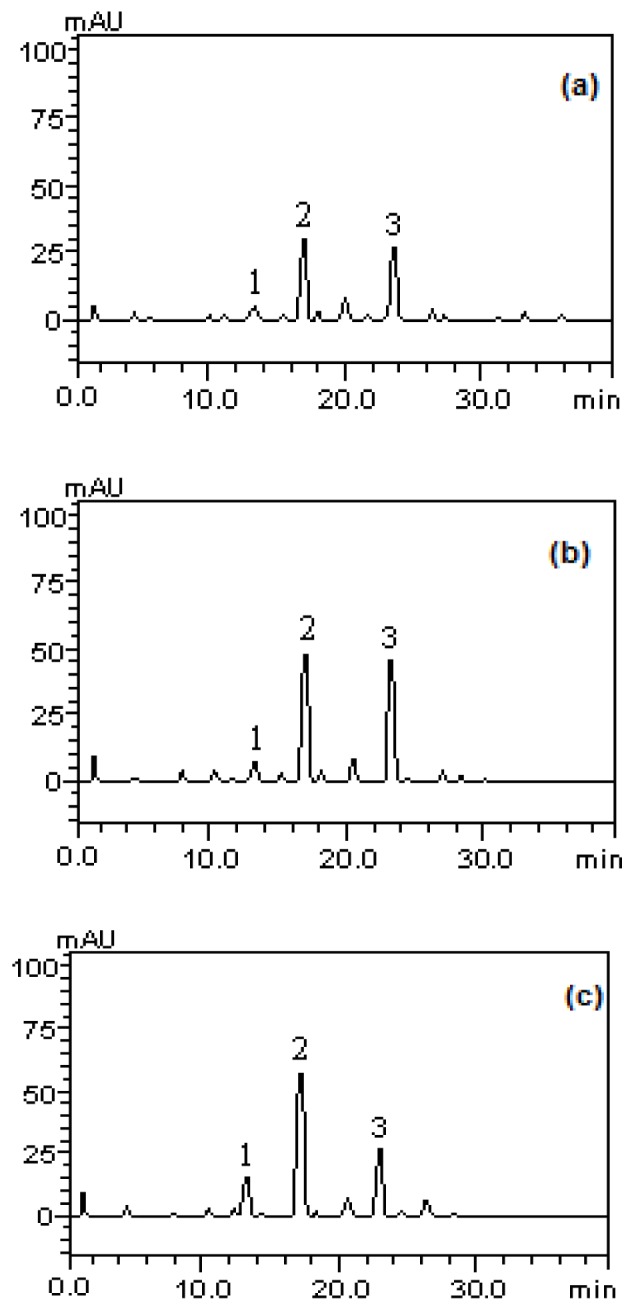
Analysis profile of the methanolic fractions of *P.guajava* fruits in stages of immature (a), partially immature (b) and mature (c) for carotenoids. tocopherol (peak 1), lycopene (peak 2), β-carotene (peak 3).

Regarding the presence of carotenoids, the analysis profile of the methanolic fractions of *P. guajava* fruits revealed the presence of tocopherol (tR = 13.65 min, peak 1), lycopene (tR = 17:34 min, peak 2) and β-carotene (tR = 24.01 min) (Figure 2(Fig. 2)). The structures of the identified compounds are depicted in Figure 3(Fig. 3).

### Antioxidant activity

#### DPPH

The methanolic fractions of *P. guajava* fruits in immature stages, partially mature stages and mature stages were able to neutralize the free radical DPPH. The concentrations for LC_50_ indicated similar values (859.33, 854.63 and 848.78 µg/mL), with no statistically significant difference between them, with better results for the mature stage.

The free radical DPPH neutralization reaction keeps a degree of proportionality to the amount of electron or proton donor compounds, the higher the donating capacity, the higher the neutralization. Phenolic compounds have in general a chemical structure composed of hydroxylic, carboxylic and carbonyl groups attached to the aromatic ring which are easily attacked by reactive species, the presence of these groups are directly linked to the power of neutralization of reactive species (van Acker et al., 1996[36]).

Cao et al. (1997[9]) correlate the amount of hydroxyl groups attached to the flavonoids basic structure with its antioxidant capacity and notes that the flavonoids with the largest number of hydroxyl has greater antioxidant potential. Gutierrez et al. (2008[17]) list the presence of flavonoids with hydroxylic multiligands (quercetin, leucocyanidin, kaempferol and guaijaverin), confirmed by the analysis in HPLC, in guava fruits. These flavonoids increase in concentration during ripening which can be related to the higher neutralizing activity of DPPH radical been observed in the mature stage.

#### Iron chelation assay

The results obtained for the chelating capacity of Fe^2+^ ions by the methanolic fractions of *P. guajava* fruits in different maturation stages are shown in Table 2(Tab. 2).

The highest percentage of chelation was found in the immature stage in the concentration of 250 mg/mL with 21.93 %, as the concentration was reduced, the percentage of chelation also decreased proportionally. Unlike the immature stage, the partially mature and mature stage had lower percentages of Fe^2+^ ions chelation. At its highest concentration, the partially mature stage showed 9.20 % as well as the mature stage which showed 6.57 %. For both, not all concentrations were capable of chelating iron ions.

The chelation of these ions by various compounds present in the methanolic fractions of *P. guajava* fruits, is associated with a protective effect against ROS, harmful to biological systems. The immature stage showed greater activity through this antioxidant route in relation to the direct neutralization of EROS observed in the test of free radical DPPH kidnapping. Disler et al. (1975[11]) showed that in mice, tannins present in tea are capable of acting as iron chelators inhibiting the intestinal absorption of the metal. Jain et al. (2003[21]) apud Gutierrez et al. (2008[17]) cites the green fruit as rich in tannins and their correlation with in vivo activity observed in rats, we can assume that most chelating activity presented by the fruit in the immature stage is due to the presence of tannins.

#### Lipid peroxidation

In the inhibition assay of the lipid peroxidation process was possible to observe the reduction of the baseline levels for concentrations of 250 mg/mL in the three stages, especially the mature stage. A wide variation of results was observed, and the antioxidant activity was not considered significant for any of the samples and tested concentrations. The data obtained is shown in Figure 2(Fig. 2).

According to reports from Gull et al. (2012[16]) the antioxidant activity of *P. guajava* fruits at different stages of maturity, harvested in three different regions, was due to the oxidation of linoleic acid induced by thiocyanate. The values obtained showed significant activity being the immature stage the best result. Although his studies involved a different methodology, comparison with the results obtained in this study is valid, since the antioxidant route being studied (inhibition of lipid peroxidation) is common to work.

## Conclusion

The pH and acidity of the pulps were determined and demonstrated the mature stage as the most alkaline. HPLC analysis of the methanolic fractions revealed the presence of gallic acid, catechin, chlorogenic acid, cfeic acid, epicatechin, rutin, quercitrin, isoquercitrin, quercetin, kaempferol and glycosylated campeferol, tocopherol, β-carotene and lycopene. The methanolic fractions of the three stages of maturation demonstrated the presence of phenolic compounds, flavonoids and ascorbic acid. The methanolic fractions of the three stages of maturation demonstrated capacity to neutralize free radical DPPH, the Mature stage showing the best results. The methanolic fractions of the three stages of maturation demonstrated ability to chelate Fe^2+^ ions, the green maturity stage showing the best result. The antioxidant test of lipid peroxidation did not show significant results.

## Figures and Tables

**Table 1:**
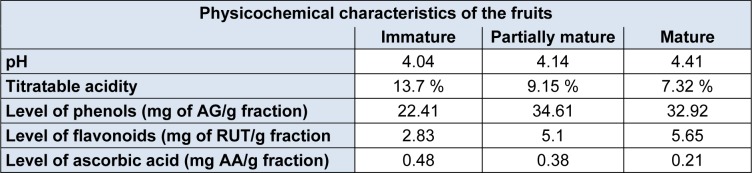
pH values, titratable acidity and levels of phenolic compounds, flavonoid compounds and ascorbic acid of *P. guajava* fruits at different stages of maturation

**Table 2:**
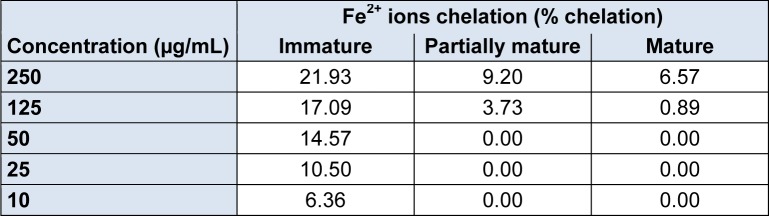
Fe^2+^ percentage chelated by the methanolic fraction of *P. guajava* fruits in the stages of immature, partially mature and mature
